# Supplementary material for: Characterization of the Community of Black Meristematic Fungi Inhabiting the External White Marble of the Florence Cathedral
Source: J Fungi (Basel). 2023 Jun 13;9(6):665. doi: 10.3390/jof9060665 (PMC10301995; doi:10.3390/jof9060665)
Supplement: Supplementary file 1 [file jof-09-00665-s001.zip › jof-2437935-supplementary.pdf]

Table S1. Sequences of the ITS and LSU regions selected from the literature belonging to fungal strains isolated from stone materials of cultural interest. The accession numbers of the sequences are indicated in the last two columns. (*) type material.

Class/Order		Strains no.	Source	Location	ITS	LSU
**Eurotiomycetes / Chaetothyriales**						
	Knufia karalitana	CCFEE 5656	marble	Santa Maria Cathedral, Cagliari (Italy)	KP791781	KR781069
	Knufia karalitana	CCFEE 5732	pietraforte (sandstone)	Boyl Palace, Cagliari (Italy)	KP791782	KR781070
	Knufia karalitana	CCFEE 5921	marble	Cemetery of Bonaria, Cagliari (Italy)	KP791784	KR781072
	Knufia marmoricola	CCFEE 5716	marble	Santa Maria Cathedral, Cagliari (Italy)	KP791786	KR781074
	Knufia marmoricola	CCFEE 5886	marble	Cortile della Pigna, Vatican City (Vatican City)	KP791779	KR781067
	Knufia marmoricola	CCFEE 5902	marble	Cortile della Pigna, Vatican City (Vatican City)	KP791776	KR781064
	Knufia perforans	CBS 885.95	marble	Temple of Apollo, Delos (Greece)	AJ244230	MH874191
	Knufia petricola	CBS 726.95*	marble	Statue, Messina (Italy)	KC978746	KC978741
	Knufia petricola	A95	marble	Statue, Messina (Italy)	KC978734	FJ358269
	Knufia petricola	CBS 725.95	calcarenite	Obelisque, Corfù (Greece)	KC978745	KC978740
	Lithohypha guttulata	CCFEE 5884	marble	Cortile della Pigna, Vatican City (Vatican City)	KP791768	KR781056
	Lithohypha guttulata	CCFEE 5885	marble	Cortile della Pigna, Vatican City (Vatican City)	KP791774	KR781062
	Lithohypha guttulata	CCFEE 5894	marble	Cortile della Pigna, Vatican City (Vatican City)	KP791769	KR781057
	Lithohypha guttulata	CCFEE 5908	marble	Cortile della Pigna, Vatican City (Vatican City)	KP791770	KR781058
**Dothideomycetes incertae sedis**						
	Coniosporium uncinatum	CBS 100219*	marble	Italia	NR145343	GU250903
	Dothideomycetes sp.	CCFEE 5945	marble	Rossino Bolla's spouses funerary monument, Cemetery of Bonaria, Cagliari (Italy)	OM568835	OM346746
**Dothideomycetes / Mycosphaerellales**						
	Vermiconia calcicola	CCFEE 5718	marble	Santa Maria Cathedral, Cagliari (Italy)	KP791762	KR781049
	Vermiconia calcicola	CCFEE 5770	marble	G. Ara funerary monument, Cemetery of Bonaria, Cagliari (Italy)	KP791759	KR781046

**Dothideomycetes / Capnodiales**	*Vermiconia calcicola*	CCFEE 5947	marble	Rossino Bolla's spouses funerary monument, Cemetery of Bonaria, Cagliari (Italy)	KP791756	KR781050
	Paradevriesia compacta	CBS 118294*	limestone	Mallorca (Spain)	NR_144955	NG_059089

Table S2. Colony morphology of the meristematic strains selected for the physiological tests. The description is referred to an incubation of 28 days on MEA.

**Strain**	**Genus**	**Description**
m8	*Knufia* sp.	colonies growing very slowly, up to 9 mm in diameter; greenish-black, raised centrally and flat near the periphery, velvety, regular margin.
m10	*Vermiconia calcicola*	colonies growing very slowly, up to 5 mm in diameter; yellowish-black, cerebriform, growth mainly vertical, velvety with dark margin.
m15	*Knufia marmoricola*	colonies growing slowly, up to 11 mm in diameter; brownish-black, vertical growth of the mycelium surface in bunches, especially in the center.
m16	*Knufia marmoricola*	colonies growing slowly, up to 14 mm in diameter; grey, moderate vertical growth centrally and even flat near the periphery, velvety, regular margin.
m20	*Lithohypha/Coniosporium*	colonies growing slowly, up to 10 mm in diameter; brownish-black, vertical growth of all the mycelium surface in bunches, velvety, irregular margin.
m21	*Lithohypha guttulata*	colonies growing slowly, up to 10 mm in diameter; grey, cauliflower-shaped, finely velvety, irregularly lobate margin.
M30	*Coniosporium uncinatum*	colonies growing very slowly, up to 7 mm in diameter; greenish-black, vertical growth of all the mycelium surface in bunches, velvety.
TW-N-Tq	*Dothideomycetes* sp.	colonies growing slowly, up to 10 mm in diameter; black, cerebriform, glossy, irregular lobate margin.

Table S3. Results of the sensitivity test to biocides after 28 days of incubation. Measures of colonies size are expressed in mm ± SD.

	control (DMSO)	EO-Origanum				EO-Thymus				BiotinT			
**Strain**	0	0.0025%	0.005%	0.012%	0.025%	0.0025%	0.005%	0.012%	0.025%	0.0025%	0.005%	0.012%	0.025%
m8	7.75 ± 2.04	4.50 ± 0.58	4.25 ± 2.63	0.00	0.00	5.17 ± 1.72	7.13 ± 3.17	3.17 ± 0.98	0.00	2.38 ± 0.48	2.50 ± 1.05	1.50 ± 0.55	1.75 ± 0.42
m10	4.75 ± 0.61	5.75 ± 1.50	5.50 ± 1.05	1.08 ± 0.80	0.00	5.25 ± 0.61	6.75 ± 0.96	5.08 ± 0.66	1.00 ± 0.00	5.00 ± 1.45	5.08 ± 0.80	4.17 ± 1.17	3.50 ± 1.52
m15	11.33 ± 1.99	5.50 ± 1.83	5.08 ± 1.91	0.00	0.00	7.17 ± 2.14	7.75 ± 1.50	4.08 ± 1.63	0.00	7.67 ± 0.88	2.33 ± 0.82	2.58 ± 0.66	1.00 ± 0.89
m16	9.67 ± 0.98	7.25 ± 1.61	7.00 ± 1.83	0.00	0.00	10.92 ± 1.11	8.50 ± 0.84	5.42 ± 1.28	0.00	6.92 ± 1.50	5.83 ± 0.68	2.67 ± 1.03	2.67 ± 1.03
m20	10.17 ± 2.64	4.75 ± 0.96	3.50 ± 1.29	0.83 ± 0.41	0.00	6.50 ± 1.52	4.67 ± 0.75	4.25 ± 1.25	0.92 ± 0.66	4.67 ± 0.52	4.67 ± 0.88	4.50 ± 0.84	4.42 ± 1.02
m21	10.50 ± 0.58	11.17 ± 3.76	12.50 ± 1.73	4.67 ± 1.21	0.00	12.25 ± 2.32	10.08 ± 1.16	10.33 ± 0.82	4.50 ± 0.63	9.83 ± 0.75	8.58 ± 0.92	7.25 ± 1.17	6.83 ± 1.94
M30	6.25 ± 1.72	6.00 ± 1.41	5.50 ± 0.84	0.00	0.00	7.25 ± 0.99	6.00 ± 0.89	3.83 ± 0.75	0.00	6.58 ± 0.49	6.50 ± 0.84	5.00 ± 0.63	4.17 ± 0.75
TW-N-Tq	8.08 ± 2.69	5.00 ± 0.82	4.58 ± 1.43	0.00	0.00	6.50 ± 0.84	7.25 ± 0.88	3.50 ± 0.84	0.00	5.08 ± 0.66	4.42 ± 0.92	2.00 ± 1.22	0.17 ± 0.41

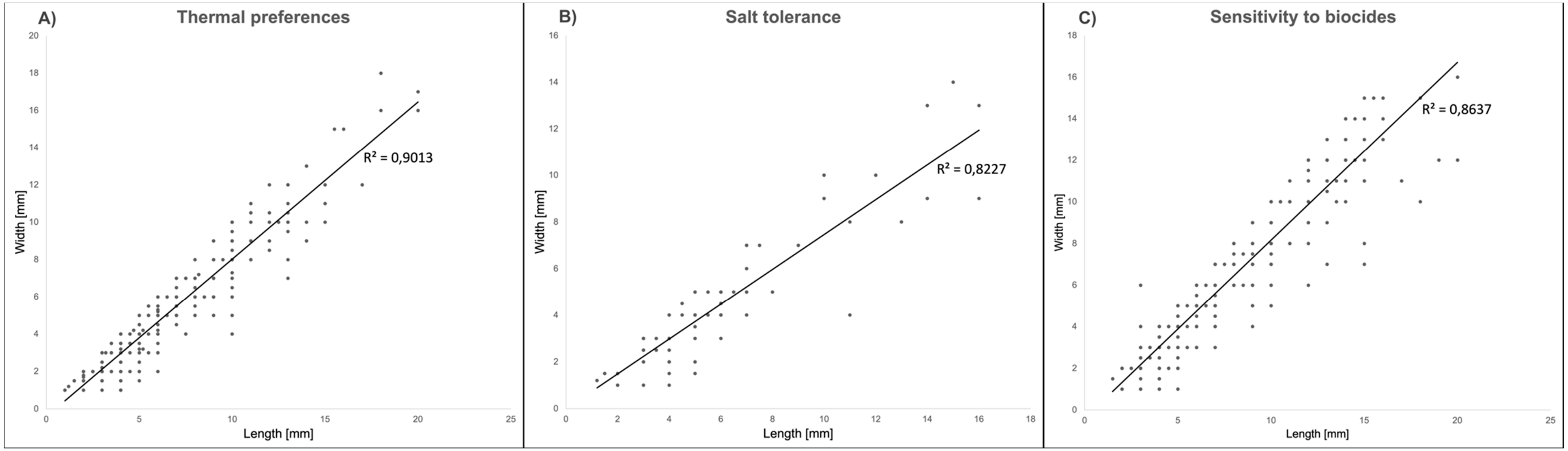

Figure S1. Linear regression between width and length measures of mycelium for the three physiological tests reported in 3.3. (A) thermal preferences; (B) salt tolerance; (C) sensitivity to biocides.

a)

Strain	Genus	acid production
m8	*Knufia* sp.	+
m10	*Vermiconia* sp.	++
m15	*Knufia* sp.	-
m16	*Knufia* sp.	++
m20	*Coniosporium* sp.	+
m21	*Lithohypha* sp.	+++
M30	*Coniosporium* sp.	+
TW-N-Tq	*Dothideomycetes* sp.	++

b)

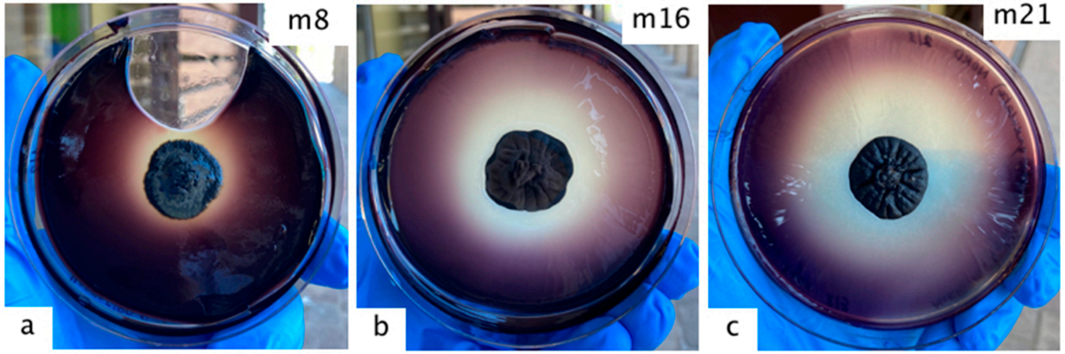

Figure S2. Results of the carbonate solubilization test. (**A**) Results of the screening expressed as: (-) negative; (+), (++) and (+++) positive response from the lowest to the highest diameter of the lysis halo around the colony; (**B**) Strains m8 (+), m16 (++) and m21 (+++) on $CaCO_3$ Agar after the treatment with Lugol's solution. The upper section of the culture medium of m8 plate (panel a) was cut due to a small contamination.
